# EVERYDAY CHALLENGES IN OLDER ADULTS WITH TRAUMATIC BRAIN INJURY: A CASE STUDY OF TWO DYADS

**DOI:** 10.1093/geroni/igae098.3968

**Published:** 2024-12-31

**Authors:** Tooba Umar, Cassie Payonk, Olivia Kuhl, Raksha Mudar, Wendy Rogers

**Affiliations:** University of Illinois Urbana Champaign, Champaign, Illinois, United States; University of Illinois Urbana Champaign, Champaign, Illinois, United States; University of Illinois Urbana Champaign, Champaign, Illinois, United States; University of Illinois Urbana Champaign, Champaign, Illinois, United States; University of Illinois Urbana Champaign, Champaign, Illinois, United States

## Abstract

Traumatic Brain Injury (TBI) results in various cognitive, somatic, and psychological challenges that affect daily life. We explored self-perceptions of these challenges in older persons with TBI (PwTBI) and their care partners (CareP), using data from two dyads (N=4). We interviewed participants about daily activities and related cognitive challenges in five domains: health, social engagement, transportation, domestic life, and leisure activities. Participants rated the difficulty of activities for the PwTBI on a scale from 1 (very easy) to 5 (very difficult). Thematic and content analyses revealed discordances in perceptions between PwTBI and CareP on several domains in both dyads, with concordance only in diet and nutrition activities in Dyad 1, which are crucial aspects of the health domain. Other activities showed discordances across both participants. In Dyad 1, PwTBI reported cognitive challenges more frequently than their CareP. However, in Dyad 2, the PwTBI reported cognitive difficulties much less frequently during activity ratings. Conversely, their care partner reported cognitive difficulties related to episodic memory and social cognition affecting the domains of health, social engagement, and transportation. These findings underscore the complexity of cognitive challenges faced by PwTBI and the differing perspectives between participants and care partners. The study highlights the need for tailored interventions that address both the challenges expressed by PwTBI and those noticed by their CareP to better support daily functioning and quality of life in older adults with TBI. This dyadic approach provides valuable insights for developing personalized care strategies.

